# Novel Creation of Three-Dimensional Virtual Reality Neuroanatomy Specimens Through Freely-Available Software

**DOI:** 10.1007/s40670-026-02860-y

**Published:** 2026-08-27

**Authors:** Diana K. Sarko, Keith D. Sudheimer

**Affiliations:** Department of Biomedical Sciences, Division of Anatomy & Neurobiology, Southern Illinois University School of Medicine, 1135 Lincoln Drive, Carbondale, IL 62901 USA

**Keywords:** Neuroanatomy, Neuroanatomy education, Medical education, Neuroscience education, 3D, Virtual reality

## Abstract

Conceptualizing the three-dimensional (3D) relationships between different brain structures represents a pervasive challenge for students learning neuroanatomy. Recent technological advances allow for the creation of adaptable, future-ready curricula and enhanced remote learning neuroanatomy resources. Here, we describe methodology for generating a 3D virtual reality brain using freely-available software.

Neuroanatomy is a challenging discipline that requires navigation of three-dimensional (3D) spatial relationships. Its complexity can lead to a dislike or fear, “neurophobia,” which ultimately results in reduced confidence in related patient management [1]. One pedagogical tool that shows great promise is the use of 3D virtual/augmented reality (AR) neuroanatomy resources to enhance student learning, remote learning opportunities, and accessibility; decrease neurophobia; and build knowledge and confidence during medical training [2, 3].

Here we describe methods for 3D reconstruction of whole brain specimens that can be deployed in any anatomy lab with minimal resources. We generated a virtual version of a brain used for instruction in our anatomy lab. The virtual brain is used for remote and asynchronous learning. The 3D brain is used in conjunction with 2D pictorial reviews with labeled gyri and sulci. The brain was obtained from a donor who provided informed consent, designating theirs as a Perpetual Donation such that “part or all of the body may be retained or permanently preserved for teaching related purposes” from the Anatomical Gift Program at the authors’ institution. These digital images are completely de-identified and used only for non-commercial, educational purposes.

3D photogrammetry scans were created using an iPhone 15 (12 megapixels; 4032 × 3024) to capture still images of the brain from various angles, elevations, and distances within 1–2 ft. Images were manually collected every 2–5° of rotation as the photographer walked around the stationary brain starting at an elevation of ~ 15° and increasing elevation by ~ 15–30° after each complete rotation (i.e., 15, 45, 60, 90°). The brain was flipped and the process was repeated with the inferior view. No assistive devices (e.g., electronic turntable, automated image collection methods) were necessary. Poor quality images were culled and 300–500 full-resolution JPEGs were uploaded into free photogrammetry software Meshroom (AliceVison), which then constructed a 3D triangular mesh surface by solving for the position and location of the camera across the picture set. The resultant stereolithography (STL) file was converted into Graphics Library Transmission Format (GLTF) and Universal Scene Descriptor (USDZ) file formats with the free 3D software Blender. The GLFT and USDZ were incorporated into a secure webpage that utilizes a model-viewer script (https://github.com/google/model-viewer), which is a client-side web component. This keeps all information necessary to display the model inside the webpage, or interact with the smart device for an AR display on the computer that accesses the webpage. Our implementation through the learning management system (D2L/Brightspace) restricts public access and restricts students from downloading the model itself, but it allows students to view the model through the webpage or AR viewers on their smart devices. The webpage routes the 3D model to either a webpage-embedded viewer or an AR viewer on the student’s smartphone. The AR experience allows students to view structures from any angle by walking around the model and to scale up small structures to any size to aid viewing.

The 3D brain model can be displayed and studied outside of the anatomy lab (Figs. 1, 2 and 3) which led to overwhelmingly positive student feedback, with respondents noting that “Neuroanatomy is very difficult to visualize and you can only spend so much time in the lab, but having something you can utilize at home makes it so much easier,” and, “This really helped me get a better visual understanding of how the brain is situated and where different structures are located, which really helps me understand how lesions produce different symptoms based on location.”

This methodology creates enduring resources that can be used to preserve both standard specimens and unique pathologies indefinitely. Wherever donor consent allows, these invaluable resources should be made freely available and openly accessible to best enable student learning.

**Fig. 1:**
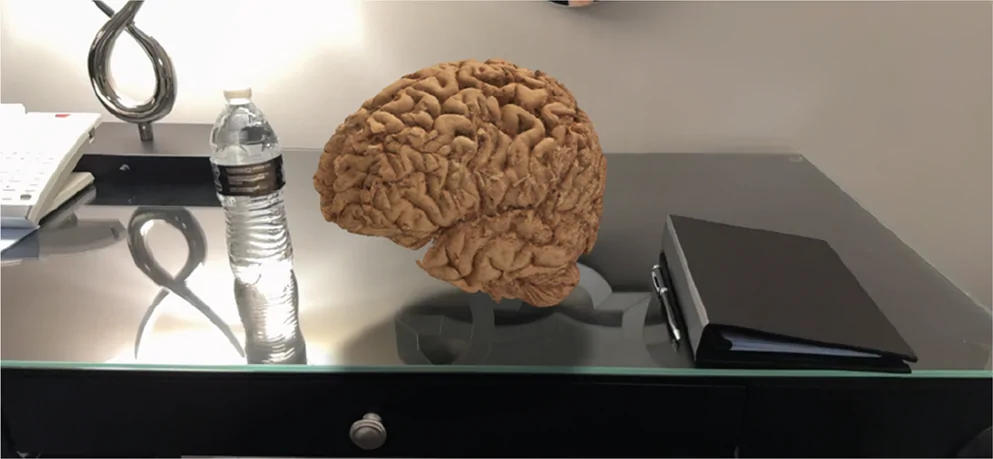
A screenshot from a mobile device AR experience. A 3D model of a postmortem human brain is displayed on a desk

**Fig. 2:**
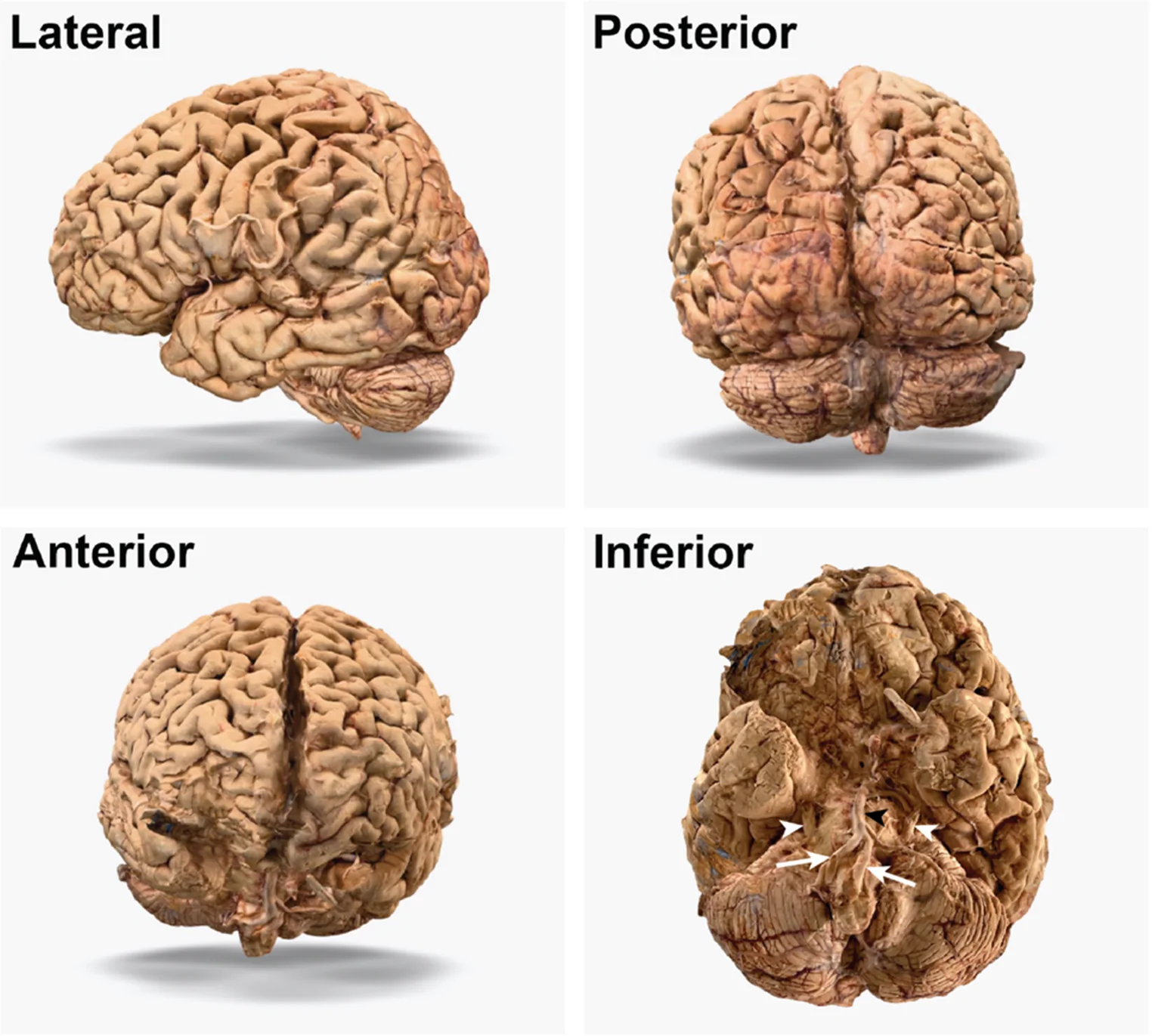
Screenshots of multiple views of the website-embedded 3D brain model. The inferior view includes the trigeminal nerves (white arrowheads) as well as the vertebral arteries (white arrows) ascending to form the basilar artery (black arrowhead)

**Fig. 3:**
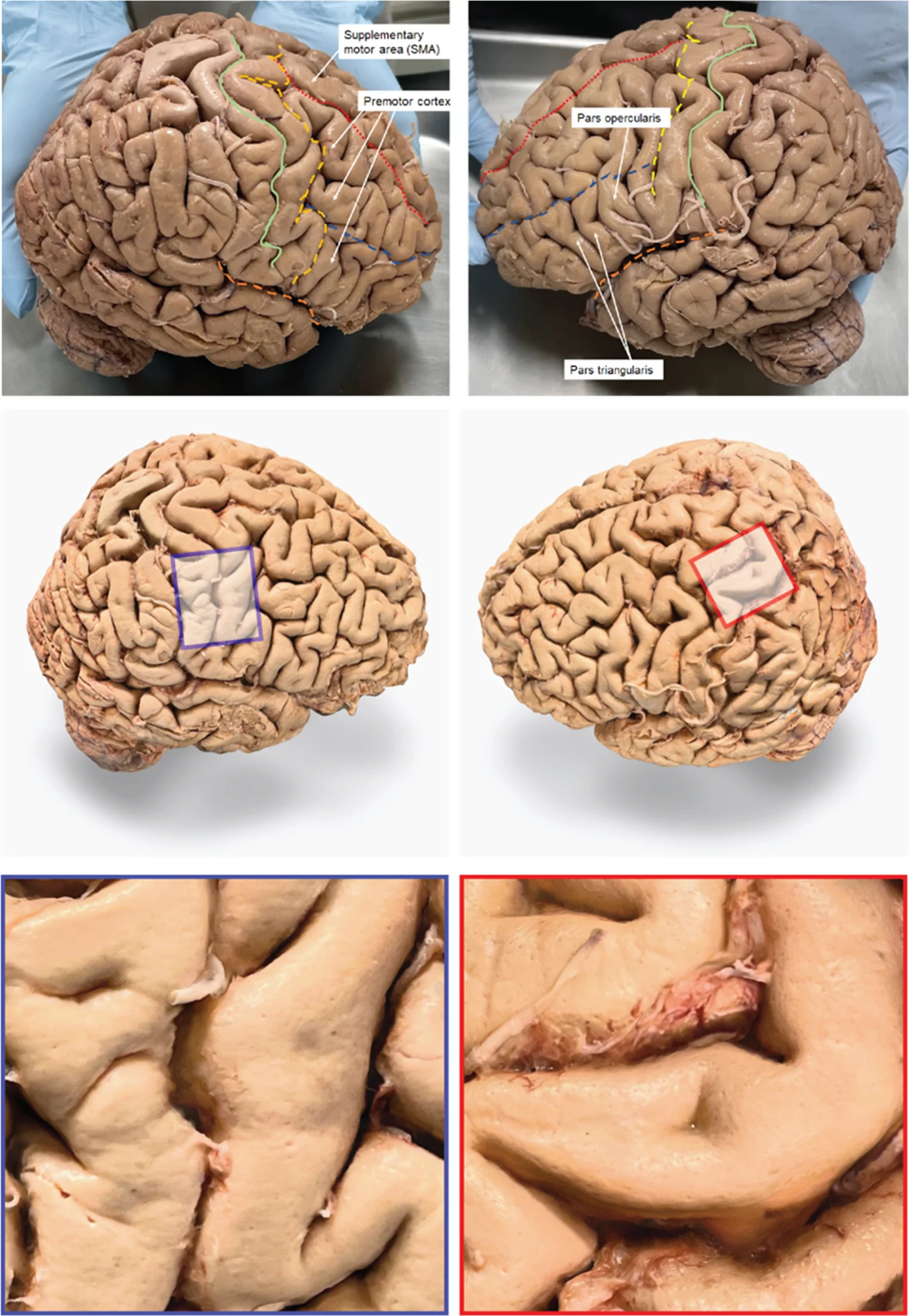
Example images from a 2D pictorial review provided to students with labeled structures and color-coded landmarks (e.g., solid green line indicating the central sulcus; top row) are shown at similar perspectives to the 3D model (middle row). Higher magnification images from these 3D models (bottom row) depict fine details of the cortical surface and vasculature. High-resolution photogrammetry scans exceed the level of detail that is directly observable in an anatomy lab without optical magnification

## References

[1] Murphy S, et al. Neurophobia amongst medical students: Hype or reality. Brain Spine. 2024;4:104134. 10.1016/j.bas.2024.104134 PMC11647157 39687082

[2] Kumar AA, et al. A 3-dimensional neuroanatomy pilot course for neurosurgical training amongst learners of differing seniorities. BMC Med Educ. 2025;25(1):1317. 10.1186/s12909-025-07901-5 PMC12492722 41039363

[3] Aridan N, et al. Neuroanatomy in virtual reality: Development and pedagogical evaluation of photogrammetry-based 3D brain models. Anat Sci Educ. 2024;17(2):239–48. 10.1002/ase.2359 37997182

